# Respiration monitoring in PACU using ventilation and gas exchange parameters

**DOI:** 10.1038/s41598-021-03639-4

**Published:** 2021-12-21

**Authors:** Hee Yong Kang, Ann Hee You, Youngsoon Kim, You Jeong Jeong, Geuk Young Jang, Tong In Oh, Yongmin Kim, Eung Je Woo

**Affiliations:** 1 Department of Anesthesiology and Pain Medicine, Kyung Hee University, Seoul, Korea; 2 Department of Biomedical Engineering, College of Medicine, Kyung Hee University, 26 Kyungheedae-ro, Dongdaemun-gu, Seoul, 02447 Korea; 3 Department of Convergence IT Engineering, POSTECH, Pohang, Korea

**Keywords:** Translational research, Respiratory signs and symptoms, Biomedical engineering

## Abstract

The importance of perioperative respiration monitoring is highlighted by high incidences of postoperative respiratory complications unrelated to the original disease. The objectives of this pilot study were to (1) simultaneously acquire respiration rate (RR), tidal volume (TV), minute ventilation (MV), SpO_2_ and PetCO_2_ from patients in post-anesthesia care unit (PACU) and (2) identify a practical continuous respiration monitoring method by analyzing the acquired data in terms of their ability and reliability in assessing a patient’s respiratory status. Thirteen non-intubated patients completed this observational study. A portable electrical impedance tomography (EIT) device was used to acquire RR_EIT_, TV and MV, while PetCO_2_, RR_Cap_ and SpO_2_ were measured by a Capnostream35. Hypoventilation and respiratory events, e.g., apnea and hypopnea, could be detected reliably using RR_EIT_, TV and MV. PetCO_2_ and SpO_2_ provided the gas exchange information, but were unable to detect hypoventilation in a timely fashion. Although SpO_2_ was stable, the sidestream capnography using the oronasal cannula was often unstable and produced fluctuating PetCO_2_ values. The coefficient of determination (R^2^) value between RR_EIT_ and RR_Cap_ was 0.65 with a percentage error of 52.5%. Based on our results, we identified RR, TV, MV and SpO_2_ as a set of respiratory parameters for robust continuous respiration monitoring of non-intubated patients. Such a respiration monitor with both ventilation and gas exchange parameters would be reliable and could be useful not only for respiration monitoring, but in making PACU discharge decisions and adjusting opioid dosage on general hospital floor. Future studies are needed to evaluate the potential clinical utility of such an integrated respiration monitor.

## Introduction

Respiratory depression and hypoventilation due to residual anesthetics and opioid administration cause hypoxemia and hypercapnia in patients in the post-anesthesia care unit (PACU) and general hospital floor (GHF), which may lead to permanent disability or life-threating complications^1–5^. Respiration monitoring would benefit non-intubated patients not only in the PACU and GHF, but during procedural sedation where Propofol is increasingly used. It would also help patients with sleep apnea and comorbidities^6,7^, obesity hypoventilation syndrome^8,9^, neuromuscular diseases^10^ and chronic obstructive pulmonary disease^11^.

In spite of a recent article reporting a pulse oximetry’s accuracy issue associated with race or skin color^12^, it is commonly used to detect hypoxemia via measuring peripheral oxygen saturation (SpO_2_). However, it often generates false or delayed alarms^5,13^. Partial pressure of end-tidal carbon dioxide (PetCO_2_) has been used to detect hypercapnia. For accurate measurements, however, it requires a stable gas sampling means, for example, an endotracheal tube or mask. Although the sidestream capnography with an oronasal cannula is used for non-intubated patients, its accuracy suffers due to the sampling tube obstruction, damping of air flow as the air passes through the tube, and cannula displacement^14,15^. A spirometer can accurately measure ventilation parameters, such as respiration rate (RR), tidal volume (TV) and minute ventilation (MV), but it may not be appropriate for continuous monitoring of spontaneously-breathing patients.

For early detection of hypoventilation in non-intubated patients, Voscopoulos et al*.*^16^ reported a respiration monitoring method using impedance plethysmography to measure RR, TV and MV. In a study with healthy volunteers, MV from impedance plethysmography showed an advantage over PetCO_2_ in tracking respiratory changes without delay^17^. Risk of hypoventilation from opioid administration increased when mild MV depression had been observed during anesthesia recovery^18,19^. MV was also able to detect respiratory compromise earlier than SpO_2_ with a smaller number of false alarms^20^. In a recent multi-center study, the utility of TV and MV as a means for perioperative respiratory status management was assessed in PACU and GHF. When two respiratory events, i.e., apnea longer than 30 s and MV below 40% of a predicted normal value, were used as actionable alarm conditions, the number of non-actionable alarms was significantly smaller than that using SpO_2_ and PetCO_2_^21^.

In spite of the advantages of ventilation parameters, however, they cannot directly detect hypoxemia and hypercapnia. Thus, an ideal respiration monitoring device should include gas exchange parameters as well as ventilation parameters. In this paper, we used two independent respiration monitoring devices to simultaneously acquire ventilation and gas exchange parameters, i.e., RR, TV, MV, SpO_2_ and PetCO_2_, from non-intubated patients in PACU. We analyzed the acquired data and assessed their capability and reliability in assessing a patient’s respiratory status and identified a practical continuous respiration monitoring method for future clinical studies.

## Methods

### Study design and data collection

Ethical approval for this study (KHUH-2019-08-058) was provided by the Institutional Review Board of Kyung Hee University Hospital, Seoul, Korea on November 11, 2019. Written informed consent forms were obtained from all patients before the study. The study was conducted between December 9, 2019 and January 23, 2020. This manuscript adheres to the strengthening the reporting of observational studies in epidemiology (STROBE) guidelines (https://www.strobe-statement.org).

In our hospital, the PACU with 10 beds is staffed by one anesthesiologist and three nurses. The clinicians use the Modified Aldrete Scoring System^22^ in assessing each patient’s conditions and deciding when the patient can be discharged from the PACU. The current practice based on the Modified Aldrete Scoring System does not include any quantitative measurements of respiratory events including apnea and hypopnea. When hypoventilation is suspected in a patient based on consciousness, spontaneous breathing efforts, and SpO_2_ values, the clinician wakes up the patient. If it fails, the oxygen supply is restarted and in the worst case tracheal intubation is performed.

**Figure 1:**
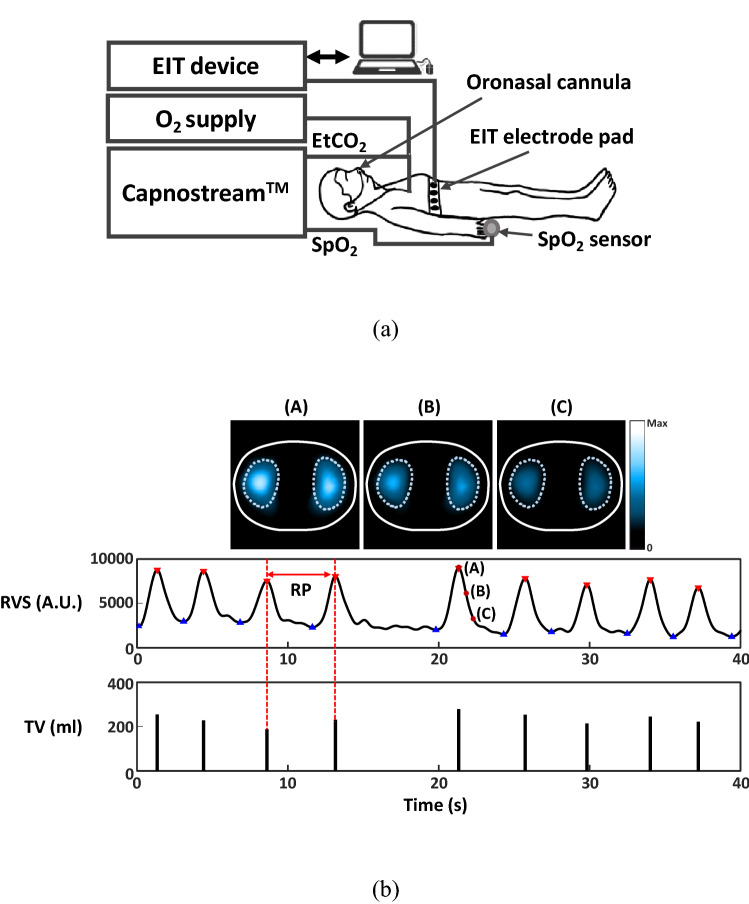
**(a)** Clinical study setup. **(b)** EIT images of lung ventilation during specific time instances of (A), (B) and (C) corresponding to the end of inspiration, the middle of expiration, and the end of expiration, respectively. The RVS was obtained as a sum of pixel values in the lung ROIs. A TV value in arbitrary units was extracted as a valley-to-peak value in the RVS. The PowerPoint (Microsoft, U.S.) and Matlab (MathWorks, U.S.) software was used to generate the diagram in **(a)** and plots in **(b)**, respectively.

In this clinical study, two continuous respiration monitoring devices were attached to each patient to collect ventilation and gas exchange parameters and other data for subsequent analysis as shown in Fig. 1a. These devices and the measured parameters except SpO_2_ were not clinically used by the clinicians in the PACU. A Capnostream35 (Medtronic, U.S.) measured the sidestream capnography signal and SpO_2_, and output PetCO_2_ and RR_Cap_ derived from the capnography signal and SpO_2_. In addition, it detected apneic events and computed the Integrated Pulmonary Index (IPI)^23^. RR_EIT_, TV and MV were measured using an electrical impedance tomography (EIT) device (AirTom-R, BiLab, Korea) originally developed for regional lung ventilation imaging^24–28^. Before a patient underwent surgery, a single electrode pad with 17 electrodes including one reference electrode was attached around the chest at the fifth intercostal space for multi-channel EIT data acquisition. This position was used to minimize the influence of diaphragm motions on the acquired EIT data^29^. The electrode pad was kept attached during surgery and postoperative monitoring in PACU.

Fifteen female patients who had been scheduled to undergo total knee arthroplasty with general anesthesia participated in this study. When the patients after surgery were transferred to PACU, the data collection began and continued throughout their PACU stay of about 60 min. During the first half of this period, 4 L/min (36% FiO_2_) of oxygen was provided through an oronasal cannula except the patient #11 who breathed with room air throughout her PACU stay. During the second half, the patient breathed spontaneously with room air.

### Data processing

The EIT device produced the reconstructed images shown in Fig. 1b at 100 frames/s using the 208-channel time-varying electrical impedance data measured around the chest. From these reconstructed EIT images, the respiratory volume signal (RVS) of relative changes in lung air volume was generated as the sum of pixel values in the regions-of-interest (ROIs) corresponding to the lungs^28,30^. This is equivalent to spatial filtering by isolating and using the variations inside the lungs while excluding other variations outside the lungs, e.g., variations due to blood flow in the heart and movements of the chest wall. Following this spatial filtering, a lowpass filter with a cutoff frequency of 0.6 Hz was applied to the RVS to remove the effects of lung perfusion, the fundamental frequency of which is higher than that of lung ventilation.

A TV value for each breathing cycle was computed in arbitrary units as the valley-to-peak value in the RVS for the corresponding breathing cycle. Before each patient was transferred from PACU to GHF, simultaneous TV measurements using EIT and a pneumotachometer-based spirometer (BTL CardioPoint-Spiro, BTL, UK) for one minute were made to convert the extracted TV values in arbitrary units to the absolute TV values in ml by using a derived scale factor. These calibrated TV values in ml are shown in Fig. 1b. Breath-by-breath RR_EIT_ was computed as the reciprocal of each breathing cycle’s duration. MV was computed as the sum of calibrated TV values during the most recent one-minute period.

### Detection of respiratory events and determination of ventilation status

**Figure 2:**
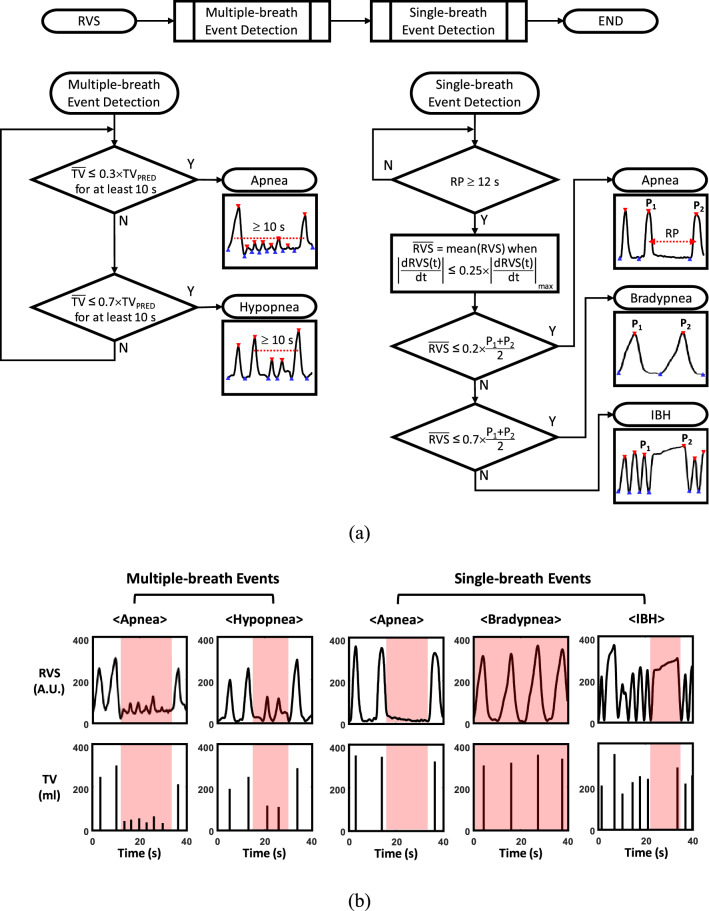
**(a)** Two-step algorithm to detect four different types of respiratory events, i.e., apnea, hypopnea, bradypnea and IBH. **(b)** Typical examples of detected respiratory events. The PowerPoint (Microsoft, U.S.) and Matlab (MathWorks, U.S.) software was used to generate the diagram in **(a)** and plots in **(b)**, respectively.

Using the RVS, TV and RR_EIT_ data, the EIT device detected four respiratory events, i.e., apnea, hypopnea, bradypnea and inspiratory breath-hold (IBH) in two steps as shown in Fig. 2a: multiple-breath event detection followed by single-breath event detection. To establish the predicted normal tidal volume (TV_PRED_) and predicted normal minute ventilation (MV_PRED_) for each patient, we used the following formulae^31,32^:

$${\mathrm{TV}}_{\mathrm{PRED}}=\left\{\begin{array}{l}6\times \left\{50.0+0.905\times \left(\mathrm{H}-152.4\right)\right\}\mathrm{ for\, a \,male\, subject }\\ 6\times \left\{45.5+0.905\times \left(\mathrm{H}-152.4\right)\right\}\mathrm{ for\, a\, female\, subject }\end{array} [\mathrm{ml}]\right.$$

and

$${\mathrm{MV}}_{\mathrm{PRED}}=\left\{\begin{array}{l}4.0\times \mathrm{BSA\, for\, a \,male\, subject }\\ 3.5\times \mathrm{BSA\, for\, a \, female\, subject }\end{array} [\mathrm{L}/\mathrm{min}]\right.$$

where H and BSA are the height in cm and body surface area in m^2^, respectively^33^.

In the multiple-breath event detection step, we first detected a respiratory event by checking $\mathrm{TV}\le 0.7\times {\mathrm{TV}}_{\mathrm{PRED}}$ for a minimum of 10 s. The 10-s duration was used following the sleep apnea scoring guideline from the American Academy of Sleep Medicine (AASM)^34^. We computed the mean TV value during the detected respiratory event ($\overline{\mathrm{TV} }$) over multiple breathing cycles. If $\overline{\mathrm{TV} }\le 0.3\times {\mathrm{TV}}_{\mathrm{PRED}}$, it was classified as apnea. Otherwise, it was classified as hypopnea.

In the single-breath event detection step, the respiration period (RP) of each breathing cycle was first computed as the time duration between two consecutive peaks in the RVS denoted as P_1_ and P_2_. If RP was longer than 12 s, we defined the breathing cycle as a long RP event. We computed the time derivative of RVS $\left(\mathrm{dRVS}(\mathrm{t})/\mathrm{dt}\right)$ during the detected long RP event and identified its sub-intervals where $\left|\mathrm{dRVS}(\mathrm{t})/\mathrm{dt}\right|\le 0.25\times {\left|\mathrm{dRVS}(\mathrm{t})/\mathrm{dt}\right|}_{\mathrm{max}}$. The mean RVS $(\overline{\mathrm{RVS} })$ was computed within the longest sub-interval. If $\overline{\mathrm{RVS} }\le 0.2\times \left({\mathrm{P}}_{1}+{\mathrm{P}}_{2}\right)$, the long RP event was classified as apnea. If $0.2\times \left({\mathrm{P}}_{1}+{\mathrm{P}}_{2}\right)<\overline{\mathrm{RVS} }\le 0.7\times \left({\mathrm{P}}_{1}+{\mathrm{P}}_{2}\right)$, it was classified as bradypnea. Otherwise, it was IBH. Figure 2b shows typical examples of the detected respiratory events by the EIT device: apnea (multiple breaths), hypopnea (multiple breaths), apnea (single breath), bradypnea and IBH.

Ventilation status was determined using the MV data measured by the EIT device. The condition of $\mathrm{MV}>{0.7\times \mathrm{MV}}_{\mathrm{PRED}}$ was used to define normal ventilation. When $\mathrm{MV}$ was less than ${0.7\times \mathrm{MV}}_{\mathrm{PRED}}$ but greater than $0.4\times {\mathrm{MV}}_{\mathrm{PRED}},$ we regarded this reduced ventilation condition as requiring watchful observation^19^. When $\mathrm{MV}$ was less than ${0.4\times \mathrm{MV}}_{\mathrm{PRED}}$, we considered it as a hypoventilation alarm condition as used by Qui et al.^21^.

The Capnostream35 detected apneas with three different durations: (1) longer than 10 s but less than 20 s, (2) longer than 20 s but less than 30 s and (3) longer than 30 s. It generated a no-breath alarm when an apnea longer than 30 s was detected. In addition, it displayed a visual alert when the number of apneas detected in an hour exceeded 10.

### Data and statistical analyses

For data analysis, we used an output file from the Capnostream35 consisting of PetCO_2_, RR_Cap_ and SpO_2_, event detection results and IPI. In addition, we used RR_EIT_, TV and MV from the EIT device. Since the Capnostream35 provided the PetCO_2_, RR_Cap_, SpO_2_ and IPI data every second, the RR_EIT_, TV and MV values corresponding to the same instances were used for analysis and plotting. We used the Matlab software (MathWorks, U.S.) for the data analyses.

The linear regression and Bland–Altman analyses were performed between RR_EIT_ and RR_Cap_. We used the modified Bland–Altman analysis for multiple replicates from each subject as pairs^35^. Different numbers of data points per subject were incorporated as weights in the analyses. We computed a 95% confidence interval (95% CI) for each of the bias and 95% limits of agreement (95% LoA). Also, we computed the percentage error (PE) between RR_EIT_ and RR_Cap_ as follows:

$$\mathrm{PE}=1.96\times \mathrm{sd}\left\{\frac{{\mathrm{RR}}_{\mathrm{EIT}}-{\mathrm{RR}}_{\mathrm{Cap}}}{\left({\mathrm{RR}}_{\mathrm{EIT}}+{\mathrm{RR}}_{\mathrm{Cap}}\right)/2}\right\}\times 100 (\%)$$

where sd{⋅} is the standard deviation function. The NCSS 2021 software (NCSS Statistical Software, U.S.) was used for the statistical analyses.

### Ethics approval

Ethical approval for this study (KHUH-2019-08-058) was provided by the Institutional Review Board of Kyung Hee University Hospital, Seoul, Korea on November 11, 2019.

### Consent to participate

Written informed consent forms were obtained from all patients before the study.

### Consent for publication

Written informed consent forms were obtained from all patients before the study.

## Results

### Observations on acquired data from 13 patients

**Figure 3:**
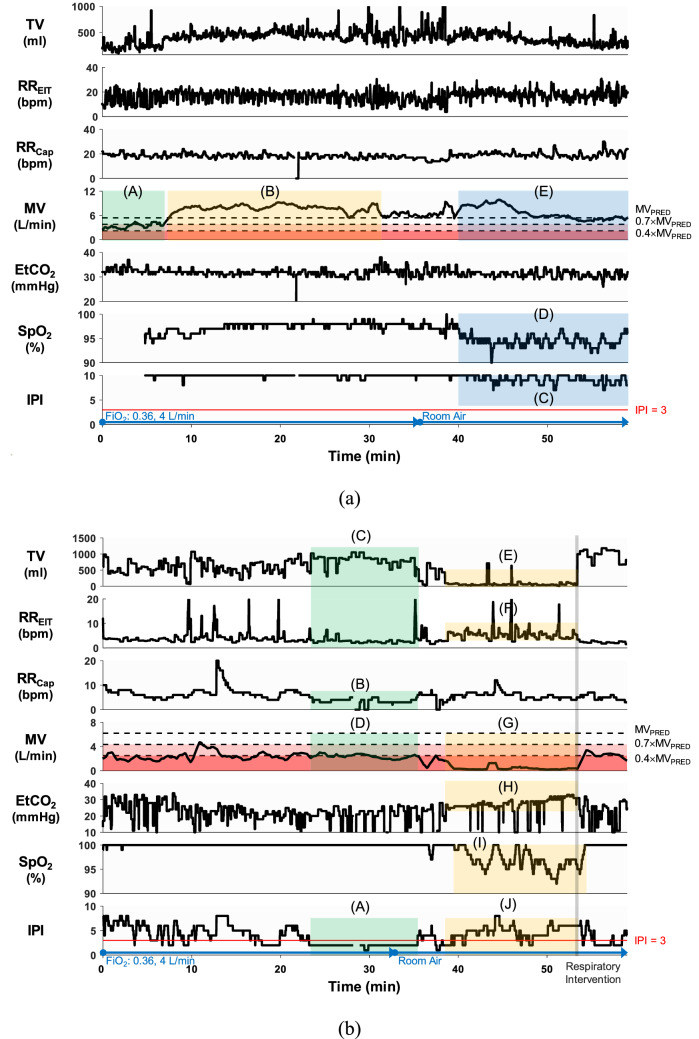

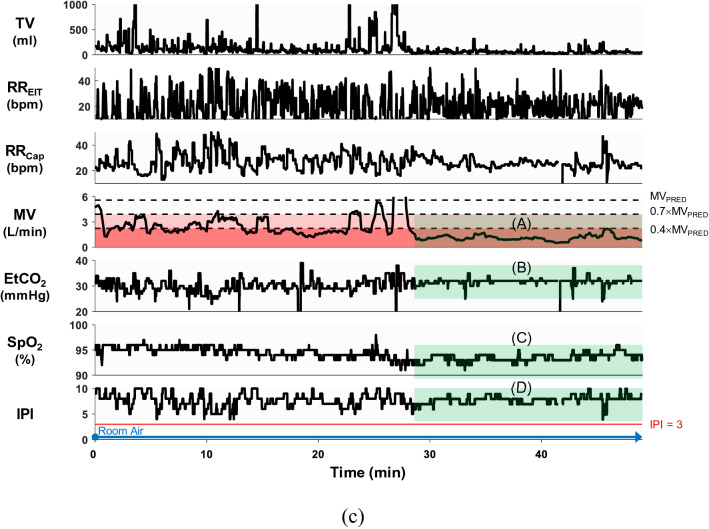
The acquired respiratory signals using the EIT device and Capnostream35. **(a)** In the patient #2, MV was low in the beginning (A), but increased to a normal level in (B). IPI fluctuated some, but stayed above the alarm threshold of 3 in (C), most likely due to SpO_2_ desaturation in (D) after switching to room air. At the end, MV was stabilized to a value around MV_PRED_ in (E). **(b)** In the patient #7, a long IPI alarm was generated in (A), likely due to the low RR_Cap_ values in (B). When RR_Cap_ dropped to 0, the IPI also showed a corresponding drop to 0. However, TV was large in (C), but RR_EIT_ remained very low. As a result, MV was on the borderline of the alarm threshold of $0.4\times {\mathrm{MV}}_{\mathrm{PRED}}$ in (D). In contrast, TV decreased significantly in (E) although RR_EIT_ was normal in (F). This combination led to greatly reduced MV values in (G), resulting in a hypoventilation episode. During this period, PetCO_2_ increased gradually in (H). The SpO_2_ fluctuation in (I) could have stemmed from reduced MV in (G). The IPI in (J) remained above 3 most of the time. The IPI alarm in (A) could have been false positive while the IPI values in (J) should have led to false negative alarm conditions. **(c)** The patient #11 breathed with room air throughout the entire measurement period. After the MV fluctuation until 28 min, the MV was stabilized to a low value below $0.4\times {\mathrm{MV}}_{\mathrm{PRED}}$, indicating a hypoventilation episode. In (B) and (C), PetCO_2_ and SpO_2_ showed a less amount of fluctuation. The IPI in (D) stayed well above the alarm threshold of 3, thus missing the hypoventilation condition in (A). The Matlab (MathWorks, U.S.) software was used to generate the plots.

Among the 15 patients, one did not complete the study due to escalated pain, and the data from another patient could not be used since the electrode pad was detached during the study. Table 1 shows the information about the remaining 13 patients who completed the study. Figure 3a is an example plot, which shows the signals from the patient #2 who had no noticeable respiratory event. The MV plot in Fig. 3 was subdivided into three regions: normal ventilation, reduced ventilation and hypoventilation. The Capnostream35’s default IPI alarm condition of $\mathrm{IPI}\le 3$ was demarcated by the horizontal red line in the IPI plot in Fig. 3. Although MV in Fig. 3a was low when this patient arrived at PACU, it increased above MV_PRED_ in 8 min and remained above MV_PRED_ for the next 40 min. MV decreased to a value near MV_PRED_ at 48 min, but stayed in the normal range until the patient left PACU in 60 min. IPI was above 8 until 40 min when it started to fluctuate between 6 and 10, most likely due to SpO_2_ desaturation after switching to room air at 35 min.

**Table 1:** Summary of patient information.

	Age (year)	Height (cm)	Weight (kg)	BSA (m^2^)	TV_PRED_ (ml)	MV_PRED_ (L/min)
#1	69	156	65	1.68	292.5	5.9
#2	74	152	56	1.54	270.8	5.4
#3	70	160	63	1.67	314.3	5.9
#4	70	156	70	1.74	292.5	6.1
#5	57	158	66	1.70	303.4	6.0
#6	69	157	72	1.77	298.0	6.2
#7	73	159	70	1.76	308.8	6.2
#8	73	149	63	1.61	254.5	5.7
#9	70	156	58	1.59	292.5	5.5
#10	67	151	55	1.52	265.4	5.3
#11	72	153	60	1.60	276.3	5.6
#12	71	150	58	1.55	260.0	5.4
#13	66	148	51	1.45	249.1	5.1

All patients were female.

*BSA* body surface area, *TV*_*PRED*_ predicted normal tidal volume for an individual patient, *MV*_*PRED*_ predicted normal minute ventilation^29,30^.

For the patient #7 in Fig. 3b, the IPI alarm was continuously turned on between 24 and 36 min, likely due to the low RR_Cap_ values during this time. However, this IPI alarm could have been false positive since the TV values during the same time period were large and the MV values were mostly above ${0.4\times \mathrm{MV}}_{\mathrm{PRED}}$. On the other hand, MV became quite low between 36 and 54 min due to the greatly reduced TV values. This caused a desaturation in SpO_2_ with a time delay of several minutes and some increase in PetCO_2_. IPI fluctuated during this 18-min time period, but mostly stayed above the alarm threshold of 3. This hypoventilation period ended by a clinician’s respiratory intervention (waking up the patient) at 54 min, resulting in the MV values increasing above ${0.4\times \mathrm{MV}}_{\mathrm{PRED}}$.

Figure 3c shows the case of the patient #11 who breathed with room air throughout the entire PACU stay. For this patient, all of the respiratory parameters fluctuated greatly during the first half. Afterwards, the amount of fluctuation decreased, but TV remained quite small, leading to a small MV value below $0.4\times {\mathrm{MV}}_{\mathrm{PRED}}$. However, IPI was above 6 most of the time between 28 and 50 min without generating an alarm. PetCO_2_ and SpO_2_ stayed at about 30 mmHg and 94%, respectively, to the end. The plots for all 13 patients are available in the online supplementary material.

### Comparison between RR_EIT_ and RR_Cap_

**Figure 4:**
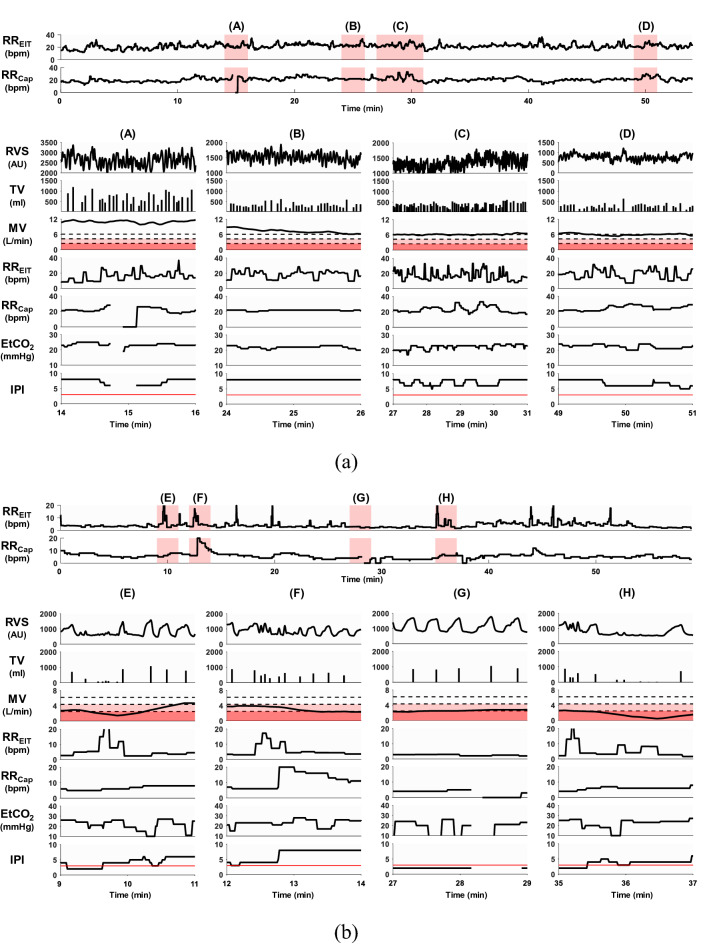
Comparison of measured ventilation and gas exchange parameters from **(a)** the patient #4 with a fast breathing pattern and **(b)** the patient #7 with a slow breathing pattern. Although RR_Cap_ and RR_EIT_ matched well for many normal breaths, RR_Cap_ was often different from RR_EIT_, e.g., (B), (C), (D), (E), (F) and (H). Sometimes, both RR_Cap_ and IPI data were missing when PetCO_2_ was unavailable, e.g., (A) and (G), likely due to the oronasal cannula misplacement or mouth breathing. The Matlab (MathWorks, U.S.) software was used to generate the plots.

The EIT device provided breath-by-breath RR_EIT_ values by detecting each breathing cycle in the RVS and was able to track both fast and slow changes in the respiration rate. Although RR_Cap_ and RR_EIT_ represented the respiration rate of the same patient and were expected to be similar, they were often different. Sometimes, both RR_Cap_ and IPI data were missing when PetCO_2_ was unavailable. In Fig. 4a and b, RR_EIT_ and RR_Cap_ were compared in patients with fast (patient #4) and slow (patient #7) breathing patterns, respectively.

**Figure 5:**
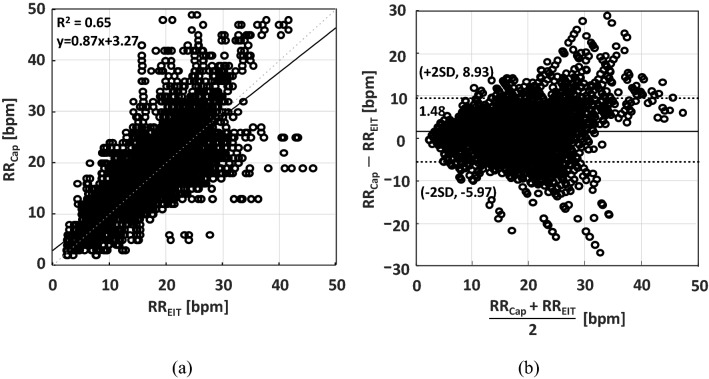
Comparison between RR_Cap_ and RR_EIT_ from all 13 patients. **(a)** Linear regression and **(b)** Bland–Altman analyses. The Matlab (MathWorks, U.S.) software was used to generate the plots.

Figure 5a shows the results of linear regression analysis using 44,513 pairs of RR_EIT_ and RR_Cap_ data pooled from all 13 patients. The coefficient of determination (R^2^) value was 0.65 between RR_Cap_ and RR_EIT_ and the p-value from a linear hypothesis test on the linear regression coefficient was 0.00. The Bland–Altman plot in Fig. 5b shows that the bias between RR_Cap_ and RR_EIT_ was 1.48 bpm with its 95% confidence interval (CI) of (−0.83, 2.13) bpm, and the 95% limits of agreement (LoA) were −5.97 and 8.93 bpm with the 95% CI of (−6.87, 9.82) bpm. The percentage error (PE) between RR_EIT_ and RR_Cap_ was 52.5%.

### Detection of respiratory events using EIT and Capnostream35

**Figure 6:**
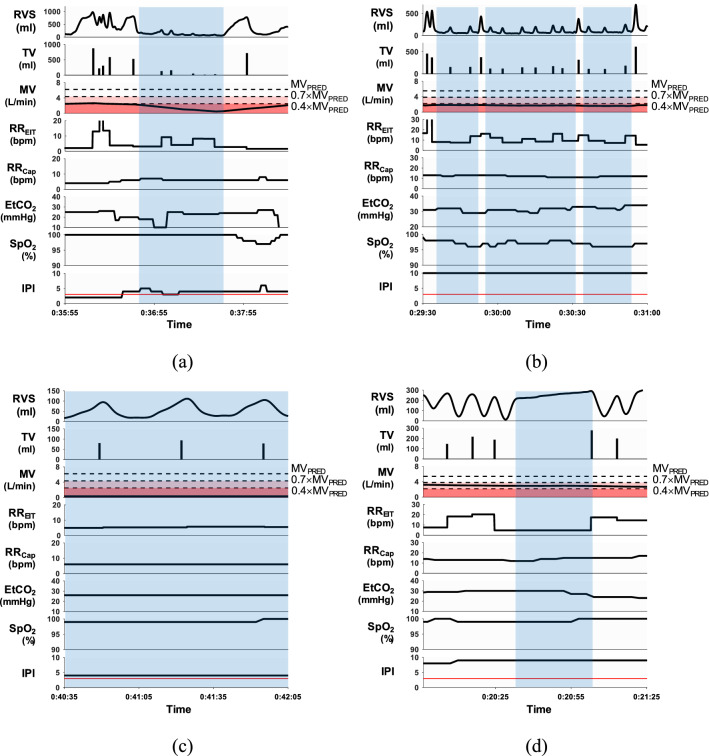
Examples of detected respiratory events: **(a)** a long apnea from the patient #7, **(b)** three consecutive hypopneas from the patient #9, **(c)** a bradypnea of three consecutive breaths from the patient #7 and **(d)** an IBH from the patient #12. Blue shaded regions indicate respiratory events detected by the EIT device. The Matlab (MathWorks, U.S.) software was used to generate the plots.

Figure 6 shows examples of detected respiratory events, and Table 2 summarizes the number of respiratory events detected by the Capnostream35 and EIT device for each patient. The Capnostream35 detected apneas longer than 10 s while the EIT device detected not only apneas longer than 10 s, but also hypopneas longer than 10 s, bradypneas with RR_EIT_ less than 5 bpm and IBHs. The number of apneas longer than 30 s is listed in the parentheses. Table 2 also includes the time duration percentage of *D*(A_Cap_), *D*(IPI), *D*(A_EIT_) and *D*(MV) corresponding to four different alarm or alert conditions, i.e., apnea detected by the Capnostream35, IPI less than or equal to 3, apnea detected by the EIT device and hypoventilation ($\mathrm{MV}\le {0.4\times \mathrm{MV}}_{\mathrm{PRED}})$. For apnea longer than 30 s, its time duration percentage is listed in the parentheses. Since the Capnostream35 did not provide the exact duration of each apnea, we considered that the duration of apnea longer than 30 s was 30 s while the duration between 20 and 29 s and between 10 and 19 s was 20 and 10 s, respectively. Therefore, *D*(A_Cap_) was underestimated to some extent.

**Table 2:** The number of apnea, hypopnea, bradypnea (RR_EIT_ ≤ 5 bpm) and inspiratory breath-hold (IBH) detected by the Capnostream35 and EIT device.

	Capnostream35			RI	EIT					
	Apnea (≥ 30 s)	*D*(A_Cap_) (≥ 30 s) in %	*D*(IPI) in %		Apnea (≥ 30 s)	Hypopnea	Bradypnea	IBH	*D*(A_EIT_) (≥ 30 s) in %	*D*(MV) in %
#1	0 (0)	0 (0)	0.2	0	0 (0)	47	15	0	0 (0)	1.22
#2	6 (2)	0.8 (0.7)	0	0	0 (0)	8	6	0	0 (0)	1.37
#3	10 (1)	1.9 (0.8)	0	0	1 (0)	1	6	0	0.5 (0)	0.41
#4	2 (1)	0.8 (0.8)	0	0	0 (0)	12	0	0	0 (0)	0.67
#5	2 (1)	0.7 (0.7)	0	0	0 (0)	64	1	0	0 (0)	60.91
#6	92 (9)	23.6 (8.3)	22.5	2	1 (1)	3	19	0	4.3 (4.3)	23.58
#7	214 (1)	28.7 (0.7)	40.2	1	3 (3)	2	112	6	21.1 (21.1)	76.11
#8	4 (1)	1.0 (0.8)	2.0	0	0 (0)	6	29	0	0 (0)	9.08
#9	4 (1)	1.2 (0.9)	0	0	0 (0)	46	4	0	0 (0)	39.89
#10	48 (1)	7.7 (0.9)	2.4	0	0 (0)	6	29	0	0 (0)	0.85
#11	4 (1)	0.9 (0.7)	0	0	31 (23)	37	3	1	52.7 (49.5)	63.32
#12	2 (1)	0.8 (0.8)	0	0	0 (0)	20	10	0	0 (0)	20.27
#13	8 (1)	1.7 (0.9)	0	0	23 (15)	21	4	0	44.0 (39.9)	86.63

The apnea alarm durations *D*(A_Cap_) and *D*(A_EIT_) were computed as the percentage time of all apneic events with respect to the total monitoring time. The number in the parentheses corresponds to the apneas longer than 30 s and their percentage time. The IPI alarm duration *D*(IPI) was computed as the percentage time with IPI ≤ 3. The MV alarm duration *D*(MV) was computed as the percentage time with MV ≤ 0.4 × MV_PRED_. RI is the number of respiratory interventions to a patient (waking up).

## Discussion

The incidence of respiratory compromise in the general care units as well as PACU could be prevented or reduced by clinicians’ timely interventions aided by an early warning system based on continuous respiration monitoring^3–5^. Recent advances in impedance plethysmography^16–21^ and EIT^24–30^ have opened up opportunities for perioperative respiration monitoring via new noninvasive tools at the bedside for continuous measurements of RR, TV and MV. Up until now, EIT devices have been mainly used specifically for determining a personalized positive end-expiratory pressure value during mechanical ventilation. Currently, they are specialized and quite expensive, which makes them unsuitable for broader clinical use. For respiration monitoring, however, a compact EIT device with a simple human interface could be developed at a fraction of the cost.

In spite of the fact that RR is one of the main vital signs that measure the body’s most basic functions, it is rarely monitored continuously in the perioperative setting^36^, primarily due to the lack of a reliable measurement tool other than a clinician’s manual counting of a patient’s breaths for 30 s or one minute. Impedance plethysmography has been used for automated measurements of RR, but its reliability was questioned, especially when physiological and/or non-physiological movements occur^37^. This is mainly due to the limited capability of a single-channel impedance plethysmography that measures a sum of all the variations caused by lung ventilation, cardiac blood flow and movements of the chest wall.

In this study on non-intubated patients with an oronasal cannula, we were able to compare RR using two different methods, i.e., RR_Cap_ and RR_EIT_. Analyzing both the RR_Cap_ and RR_EIT_ data from all 13 patients, we found a strong linear relation between them (p-value of 0.00). However, the R^2^ value was 0.65 as shown in Fig. 5a, which was surprisingly low considering that RR_Cap_ and RR_EIT_ both represented the respiration rate of an individual patient and should be nearly identical. In addition, the PE was very large (52.5%).

We found from our data on 13 non-intubated patients in PACU that the RR_EIT_ derived from the RVS was able to track fast and slow changes in breathing patterns reliably. This robustness could have stemmed from the aforementioned spatiotemporal filtering of the 208-channel EIT data in contrast to simple frequency filtering used in the conventional single-channel impedance plethysmography. However, PetCO_2_ and therefore RR_Cap_ at times failed to track changes in breathing patterns and were unavailable for a certain period of time. Thus, the discrepancy between RR_Cap_ and RR_EIT_ is more likely caused by the Capnostream35. Reasons for this could include the instability and inaccuracy of the sidestream capnography signal using an oronasal cannula due to coughing, talking and other motions in addition to cannula misplacement and moisture accumulation inside^14,15,36^. Although capnography is considered to be very reliable for continuous RR measurements on patients with masks^38^, RR measurements using the sidestream capnography with an oronasal cannula could produce erroneous results at times.

Regarding the apnea detection by the Capnostream35 and EIT, most of the Capnostream35-detected apneic events could not be found in direct ventilation measurements of RVS, TV and RR_EIT_. Comparing the number of apneas detected by the Capnostream35 and the EIT device and the values of *D*(A_Cap_) and *D*(A_EIT_) in Table 2, the number of short apneas less than 30 s detected by the Capnostream35 was much larger than that detected by the EIT device. Furthermore, many of EIT-detected long apneas greater than 30 s, e.g., in patients #11 and #13, were missed by the Capnostream35.

As an example, the EIT device was able to detect unambiguously a long apnea from the patient #7 based on breath-by-breath TV and RR_EIT_ as shown in Fig. 6a while this long apnea was missed by the Capnostream35. In Fig. 6b, three consecutive hypopneas from the patient #9 were easily detected by the EIT device. On the other hand, the Capnostream35 does not offer the capability of hypopnea detection, and its RR_Cap_ in Fig. 6b did not account for the small breaths during hypopneas. Also, since $\mathrm{MV}\le {0.4\times \mathrm{MV}}_{\mathrm{PRED}}$ represents a hypoventilation condition, the IPI value of 10 in Fig. 6b appears to be unreasonable. Examples of bradypnea and IBH are shown in Fig. 6c,d, respectively, the clinical use of which could be explored in the future.

Based on the EIT device’s apnea detection, many of those short apneas detected by the Capnostream35 were false positive, e.g., in the patients #6, #7 and #10, while the missed long apneas were false negative cases. Considering the potential danger of a long apneic event, especially in patients with comorbid sleep breathing disorders, the accurate detection of apnea longer than 30 s is clinically important^21^. Continuous measurements of TV and RR could be more advantageous than the sidestream capnography using an oronasal cannula in reliably detecting respiratory events such as apnea and hypopnea.

In Table 2, *D*(IPI) was much smaller than *D*(MV), especially for the patients #5, #7, #9, #11, #12 and #13. Based on the EIT-based hypoventilation assessment, IPI was not able to detect many hypoventilation events. Since IPI was derived from frequently inaccurate values of PetCO_2_ and RR_Cap_, the IPI value in our study often did not reflect a patient’s respiratory status correctly. Although SpO_2_ played a role in determining the IPI value^23^, the time delay associated with SpO_2_ desaturation in response to decreased MV made IPI less sensitive to hypoventilation. For reliable detection of hypoventilation, continuous measurements of MV could be more advantageous than the combination of the sidestream capnography using an oronasal cannula and SpO_2_.

In this study, we showed the future possibility of enhanced respiration monitoring with both ventilation and gas exchange parameters. We found that the ventilation parameters of RR_EIT_, TV and MV were reliable and capable of accurate and timely detection of hypoventilation and respiratory events. SpO_2_ provided oxygen desaturation information after switching to room air and during hypoventilation events in spite of its inherent delay. On the other hand, the role of capnography as a means to detect respiratory events in our study was limited due to the superior capability of the ventilation parameters. For future studies, therefore, we suggest that RR, TV, MV and SpO_2_ be used for practical continuous respiration monitoring of non-intubated patients. When hypercapnia detection is specifically desired, e.g., during oxygen therapy, however, a separate capnography device could be used.

This study with a small number of only female subjects undergoing total knee arthroplasty was intended to identify a practical continuous respiration monitoring method using both ventilation and gas exchange parameters before future clinical studies are performed to evaluate the selected method in comparison with the current practice at the center. Since no study outcomes and end points were used in this study, the results should be interpreted as only showing the early potential for respiration monitoring incorporating both ventilation and gas exchange parameters.

Although we were able to detect incidences of hypoventilation and respiratory events from the 13 non-intubated patients, there was no adverse event observed in our case series study in PACU. Large-scale studies with a diverse patient population are needed to determine the clinical feasibility and utility of perioperative respiration monitoring. Also, the total monitoring period should be extended in future studies to cover a patient’s stay both in PACU and on GHF so that cases of adverse events would be more likely to be captured. For future clinical applications, a single integrated respiration monitor with RR, TV, MV and SpO_2_ could be developed and used.

## Conclusion

In this pilot study with 13 PACU patients, we collected both ventilation and gas exchange parameters and other data, e.g., IPI, and subsequently analyzed them in detecting incidences of hypoventilation, oxygen desaturation and respiratory events. We found that continuous respiration monitoring of non-intubated patients could be performed reliably using RR, TV, MV and SpO_2_ whereas a capnographic device with an oronasal cannula is more vulnerable to artifacts due to cannula displacement, moisture in the cannula, coughing, talking and other head motions. Future studies are needed to evaluate the clinical usefulness of a respiration monitor using RR, TV, MV and SpO_2_ for respiration monitoring and making PACU discharge decisions compared with the currently-practiced PACU discharge criteria.

## Supplementary Information

Supplementary Figures.

## Data Availability

The data that support the findings of this study are available from the corresponding author, EJW, upon reasonable request.
