# Supplementary material for: Respiration monitoring in PACU using ventilation and gas exchange parameters
Source: Sci Rep. 2021 Dec 21;11:24312. doi: 10.1038/s41598-021-03639-4 (PMC8692466; doi:10.1038/s41598-021-03639-4)
Supplement: Supplementary file 1 — Supplementary Figures. [file 41598_2021_3639_MOESM1_ESM.pdf]

Supplementary Material

for

Respiration monitoring in PACU using ventilation and gas exchange parameters

Hee Yong Kang,[1] Ann Hee You,[1] Youngsoon Kim,[1] You Jeong Jeong,[2] Geuk Young Jang,[2] Tong In Oh,[2] Yongmin Kim,[3] and Eung Je Woo[2]

[1]Department of Anesthesiology and Pain Medicine, Kyung Hee University, Seoul, Korea
[2]Department of Biomedical Engineering, Kyung Hee University, Seoul, Korea
[3]Department of Convergence IT Engineering, POSTECH, Pohang, Korea

Corresponding author:

Eung Je Woo, PhD
Department of Biomedical Engineering
College of Medicine
Kyung Hee University
26 Kyungheedae-ro, Dongdaemun-gu
Seoul 02447, Korea.
Phone: +82-2-961-9502
Fax: +82-2-958-2889
Email: ejwoo@khu.ac.kr

Measured data from all 13 patients

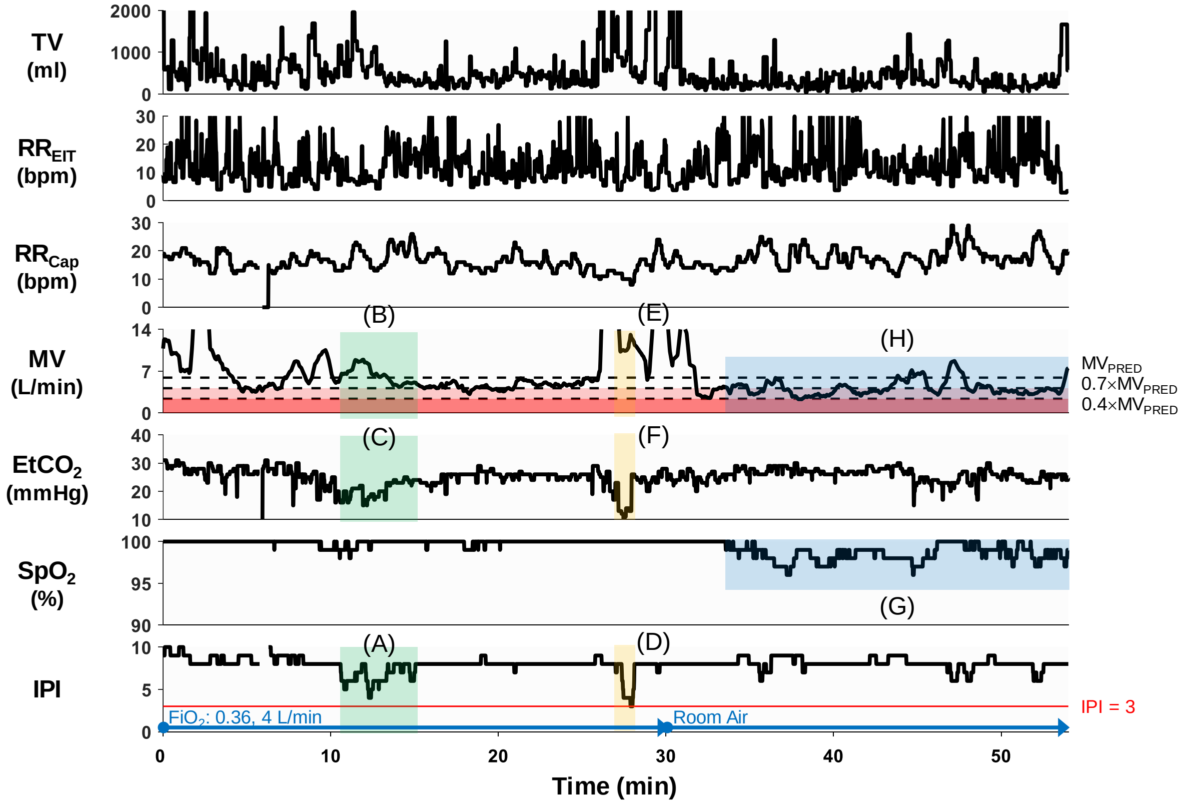

(A) IPI fluctuated between 4 and 8.

(B) MV was normal.

(C) $EtCO_2$ decreased below 20 mmHg.

(D) IPI dropped to 3 or 4.

(E) MV showed hyperventilation.

(F) $EtCO_2$ dropped to as low as 10 mmHg.

(G) SpO_2 fluctuated between 95% and 100% after switching to room air.

(H) MV decreased after switching to room air, but stayed above $0.4 \times \mathrm{MV_{PRED}}$.

Figure 1. Measured data from the patient #1. The Matlab (MathWorks, U.S.) software was used to generate the plots.

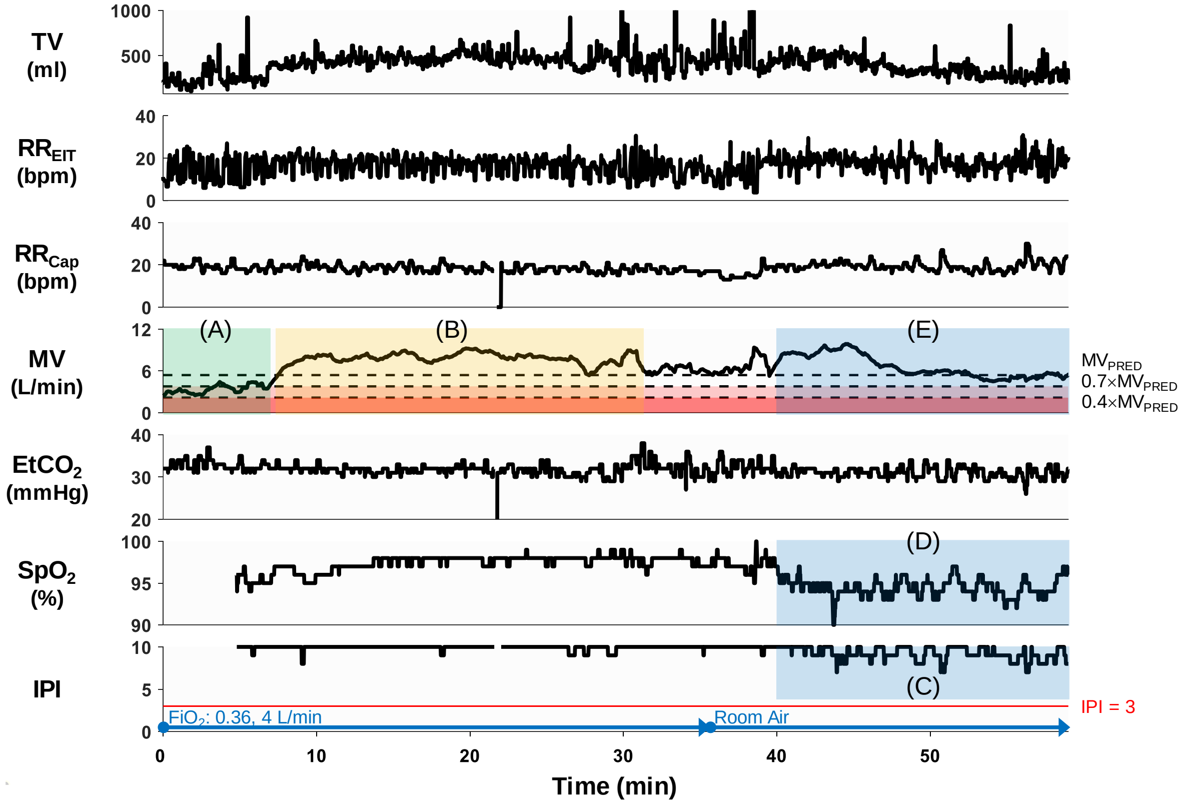

(A) MV was relatively low.

(B) MV increased above MV_{PRED} (possibly hyperventilation).

(C) IPI fluctuated between 6 and 10.

(D) SpO_2 fluctuated between 90% and 96% after switching to room air.

(E) MV decreased to a normal value.

Figure 2. Measured data from the patient #2. The Matlab (MathWorks, U.S.) software was used to generate the plots.

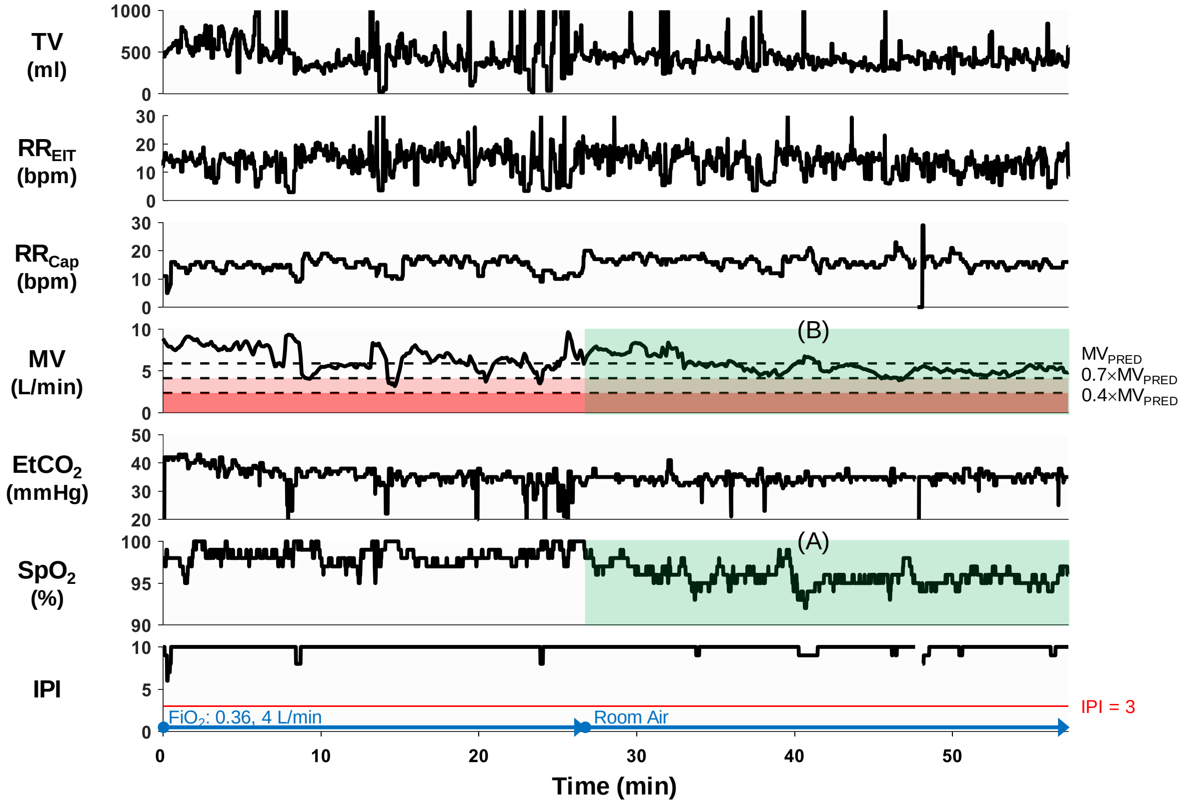

(A) SpO_2 fluctuated between 93% and 99% after switching to room air.

(B) MV decreased somewhat, but stayed within the normal range.

Figure 3. Measured data from the patient #3. The Matlab (MathWorks, U.S.) software was used to generate the plots.

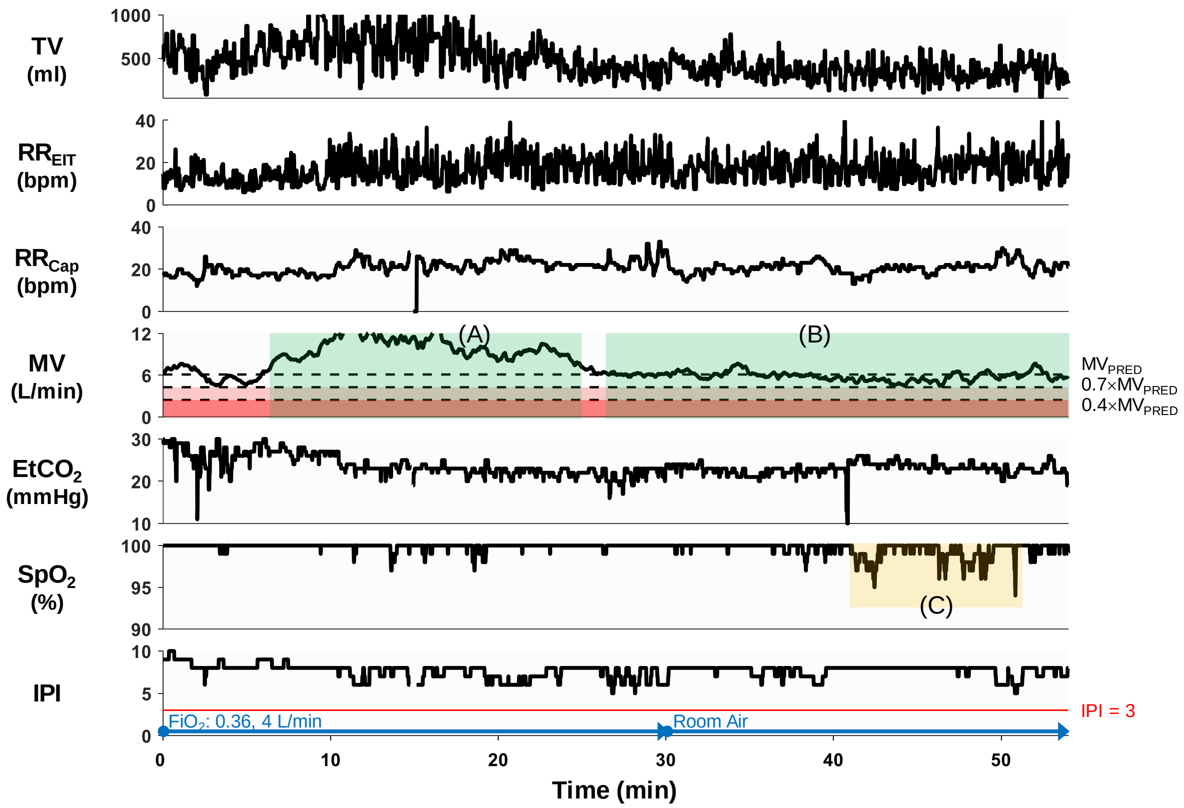

(A) MV was large (possibly hyperventilation).

(B) MV returned to normal.

(C) SpO_2 fluctuated between 94% and 100% after switching to room air.

Figure 4. Measured data from the patient #4. The Matlab (MathWorks, U.S.) software was used to generate the plots.

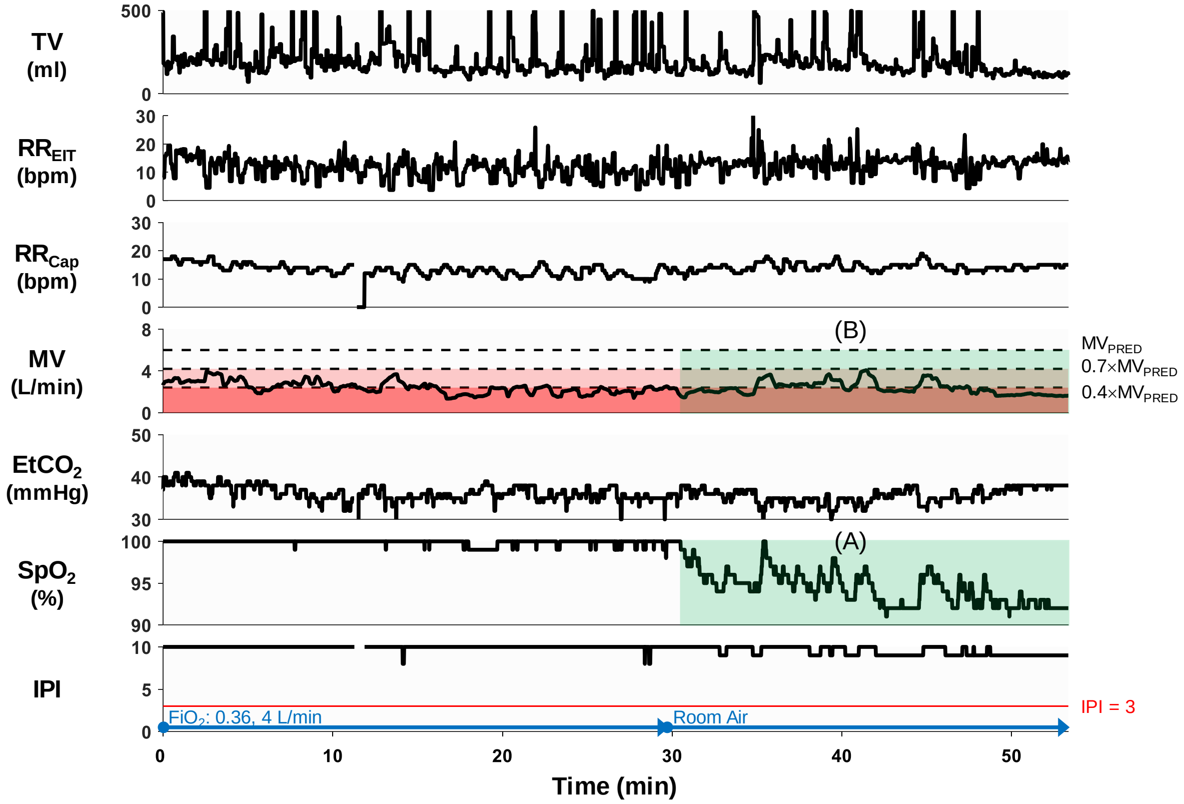

(A) SpO_2 fluctuated between 91% and 100% after switching to room air.

(B) MV stayed around $0.4 \times MV_{PRED}$.

Figure 5. Measured data from the patient #5. The Matlab (MathWorks, U.S.) software was used to generate the plots.

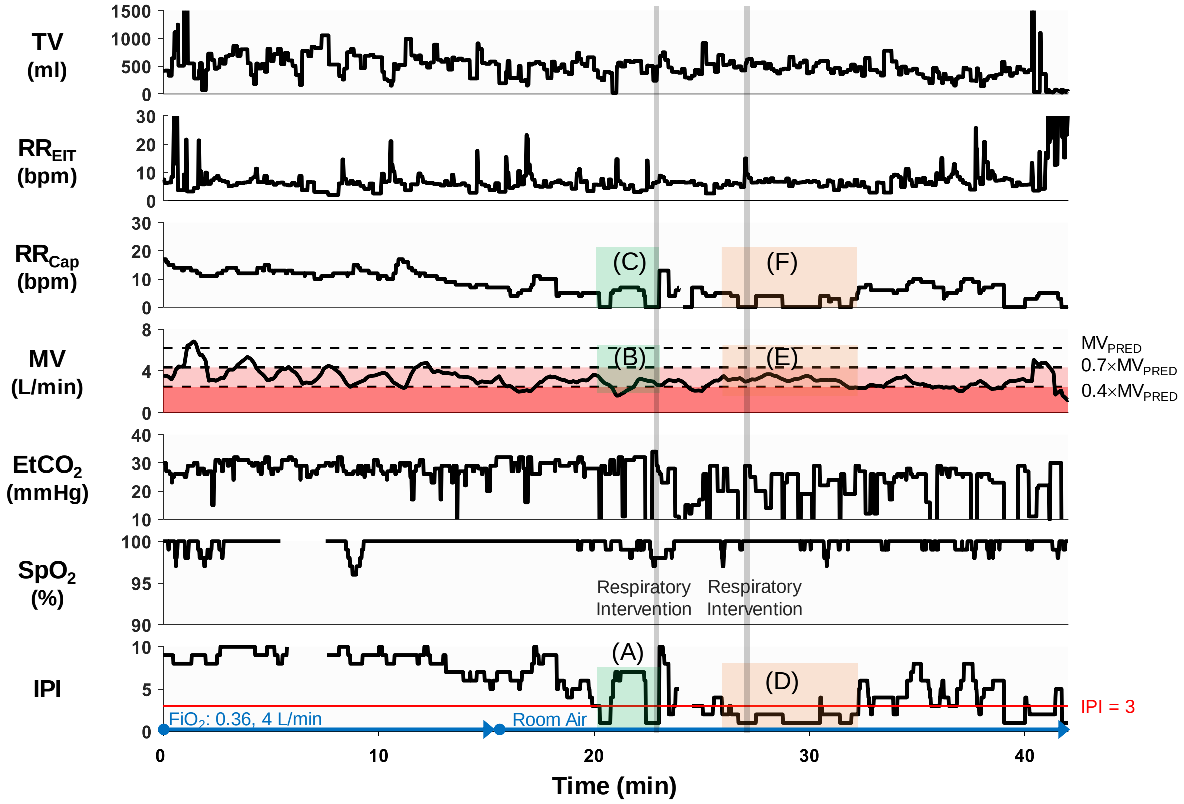

(A) IPI dropped suddenly to 1 twice.

(B) MV was around $0.4 \times MV_{PRED}$.

(C) RR_{Cap} suddenly dropped to 0 twice (we do not know the exact reason, but it is likely due to the low-quality sidestream capnography signal).

(D) IPI dropped suddenly to 1 three times and remained below 3.

(E) MV was above $0.4 \times MV_{PRED}$, but below $0.7 \times MV_{PRED}$.

(F) RR_{Cap} suddenly dropped to 0 three times.

Figure 6. Measured data from the patient #6. The Matlab (MathWorks, U.S.) software was used to generate the plots.

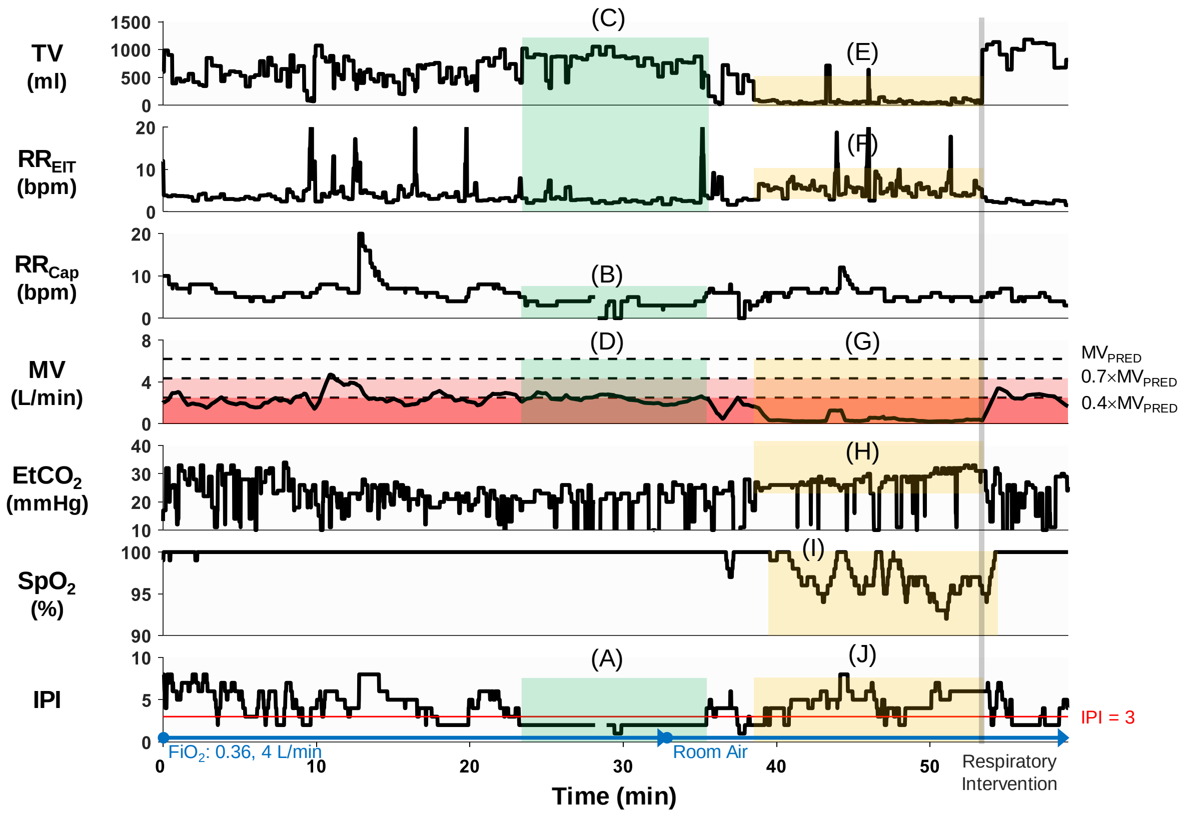

(A) IPI remained below 3.

(B) RR_{Cap} was low (we do not know the exact reason, but it is likely due to the low-quality sidestream capnography signal).

(C) TV was large, but RR_{EIT} remained very low.

(D) MV was around $0.4 \times MV_{PRED}$.

(E) TV decreased.

(F) RR_{EIT} was normal.

(G) MV decreased below $0.4 \times MV_{PRED}$ (hypoventilation).

(H) $EtCO_2$ gradually increased.

(I) SpO_2 fluctuated between 92% and 100% due to hypoventilation after switching to room air.

(J) IPI fluctuated between 2 and 10, but stayed above 3 most of the time.

Figure 7. Measured data from the patient #7. The Matlab (MathWorks, U.S.) software was used to generate the plots.

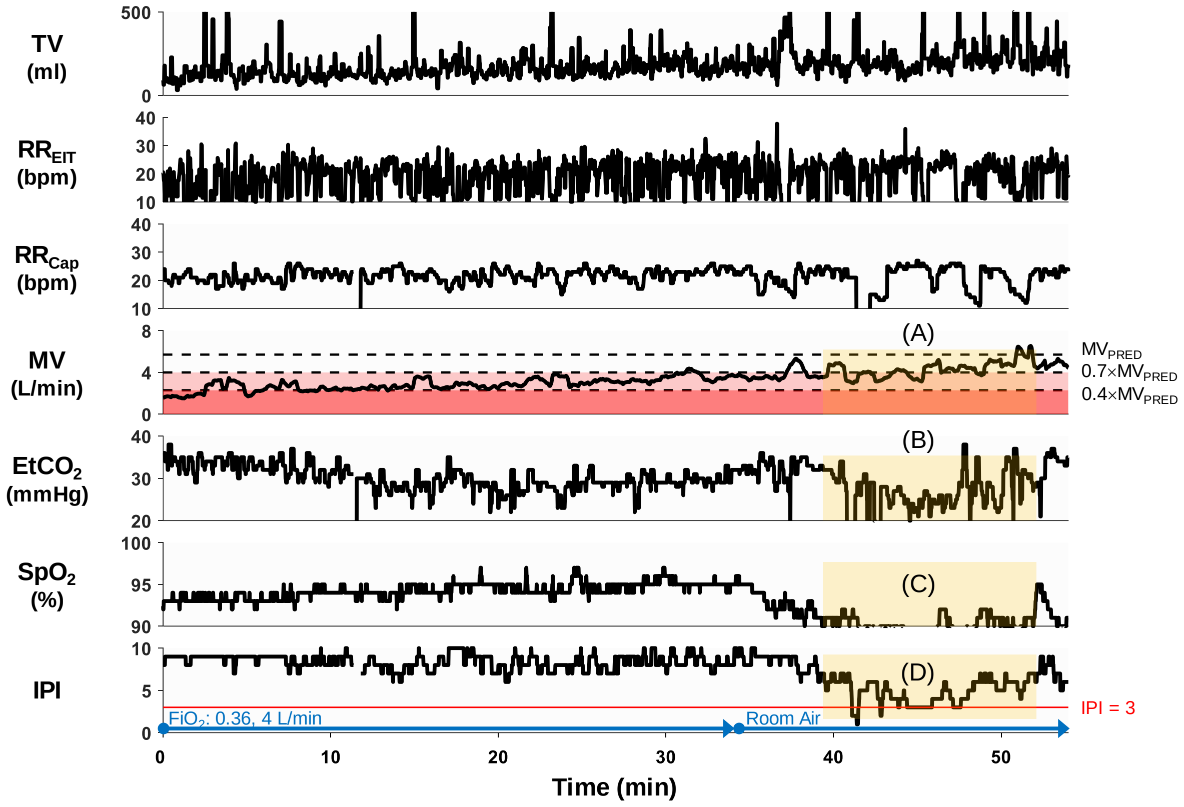

(A) MV was normal.

(B) $EtCO_2$ decreased to about 25 mmHg.

(C) SpO_2 decreased below 90%.

(D) IPI fluctuated between 1 and 7, but remained above 3 most of the time.

Figure 8. Measured data from the patient #8. The Matlab (MathWorks, U.S.) software was used to generate the plots.

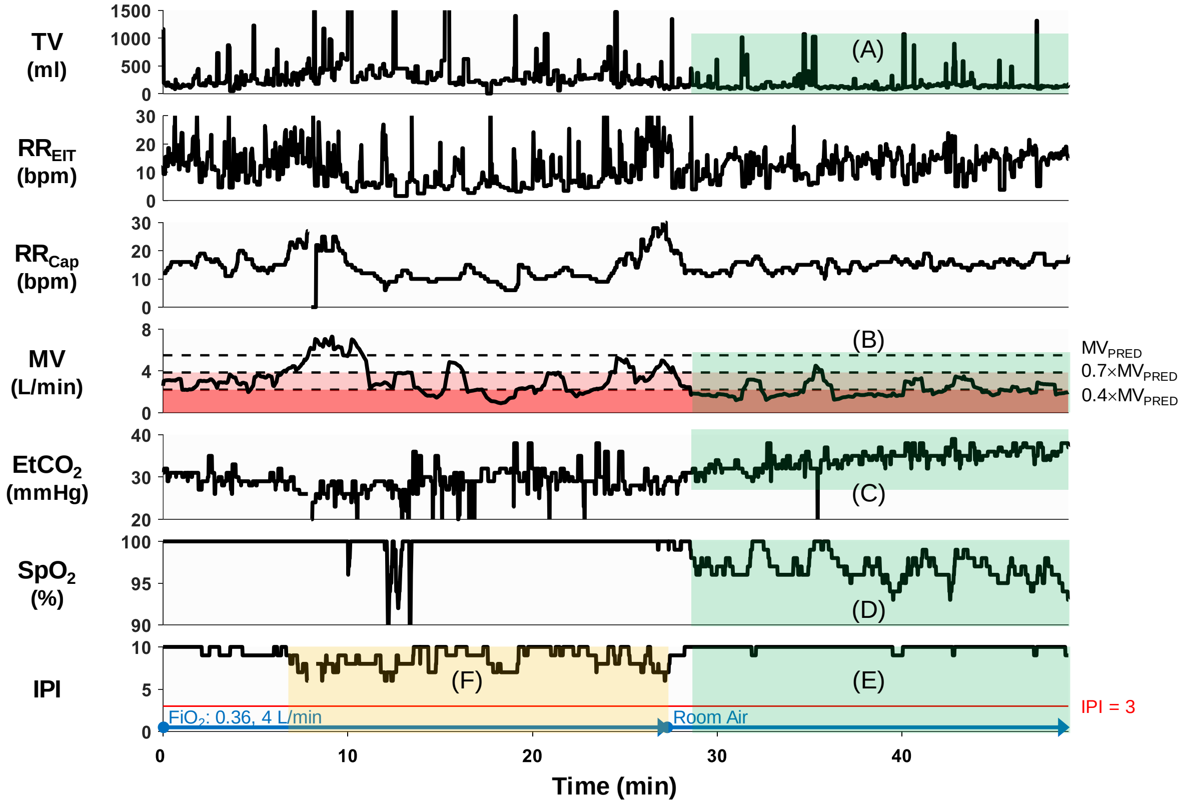

(A) TV was low with occasional deep breaths.

(B) MV was often around $0.4 \times MV_{PRED}$.

(C) $EtCO_2$ gradually increased, but remained below 40 mmHg.

(D) SpO_2 fluctuated between 94% and 100% after switching to room air.

(E) IPI was either 9 or 10.

Figure 9. Measured data from the patient #9. The Matlab (MathWorks, U.S.) software was used to generate the plots.

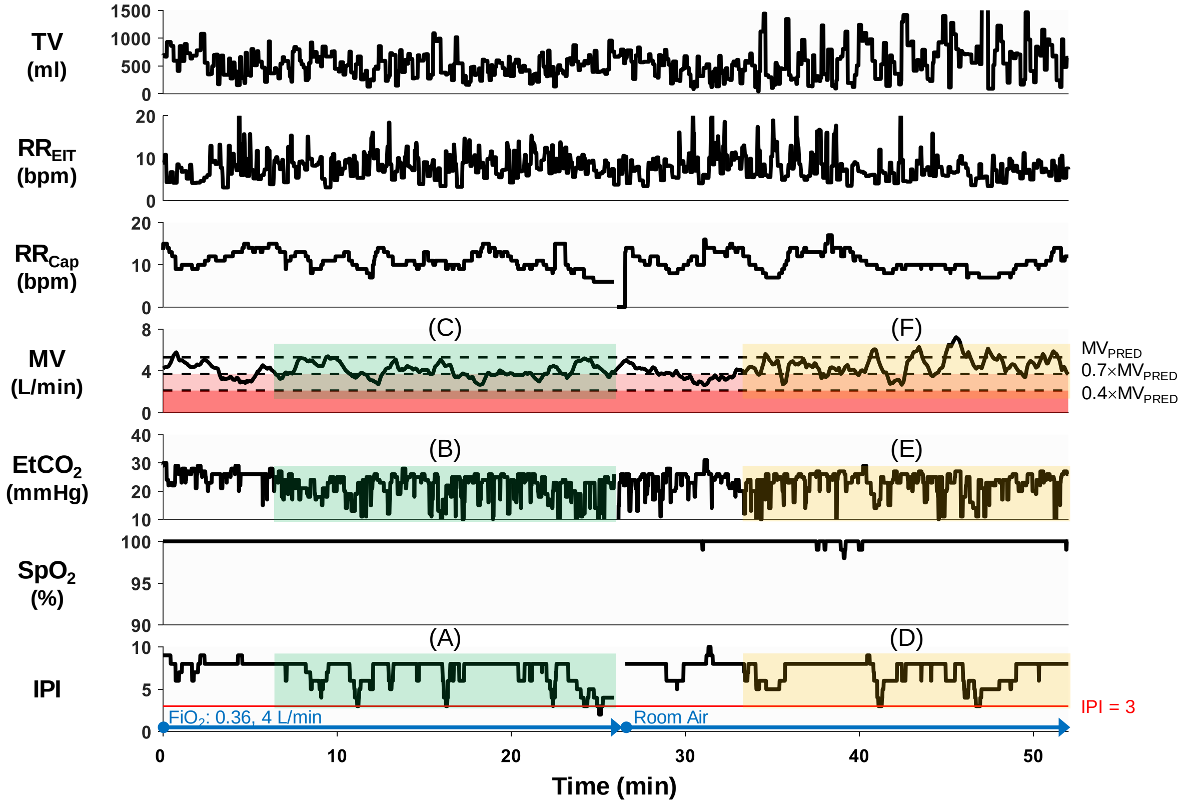

(A) IPI fluctuated between 2 and 8. The lowest IPI value of 2 occurred at around 25 minutes.

(B) $EtCO_2$ fluctuated and remained below 30 mmHg.

(C) MV was normal.

(D) IPI fluctuated between 3 and 9.

(E) $EtCO_2$ fluctuated and remained below 30 mmHg.

(F) MV was normal.

Figure 10. Measured data from the patient #10. The Matlab (MathWorks, U.S.) software was used to generate the plots.

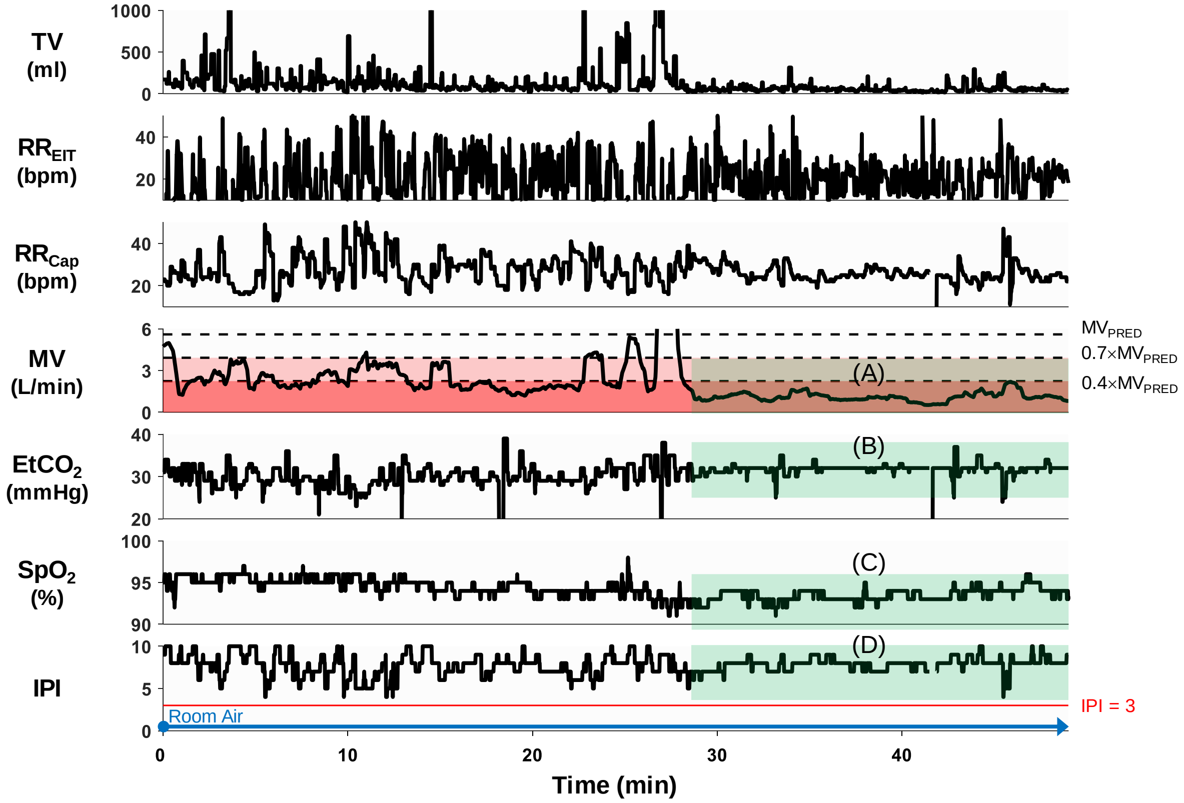

(A) MV was below $0.4 \times MV_{PRED}$ (hypoventilation).

(B) $EtCO_2$ was stable at about 33 mmHg.

(C) SpO_2 was between 91% and 96%.

(D) IPI fluctuated between 4 and 9.

Figure 11. Measured data from the patient #11. The Matlab (MathWorks, U.S.) software was used to generate the plots.

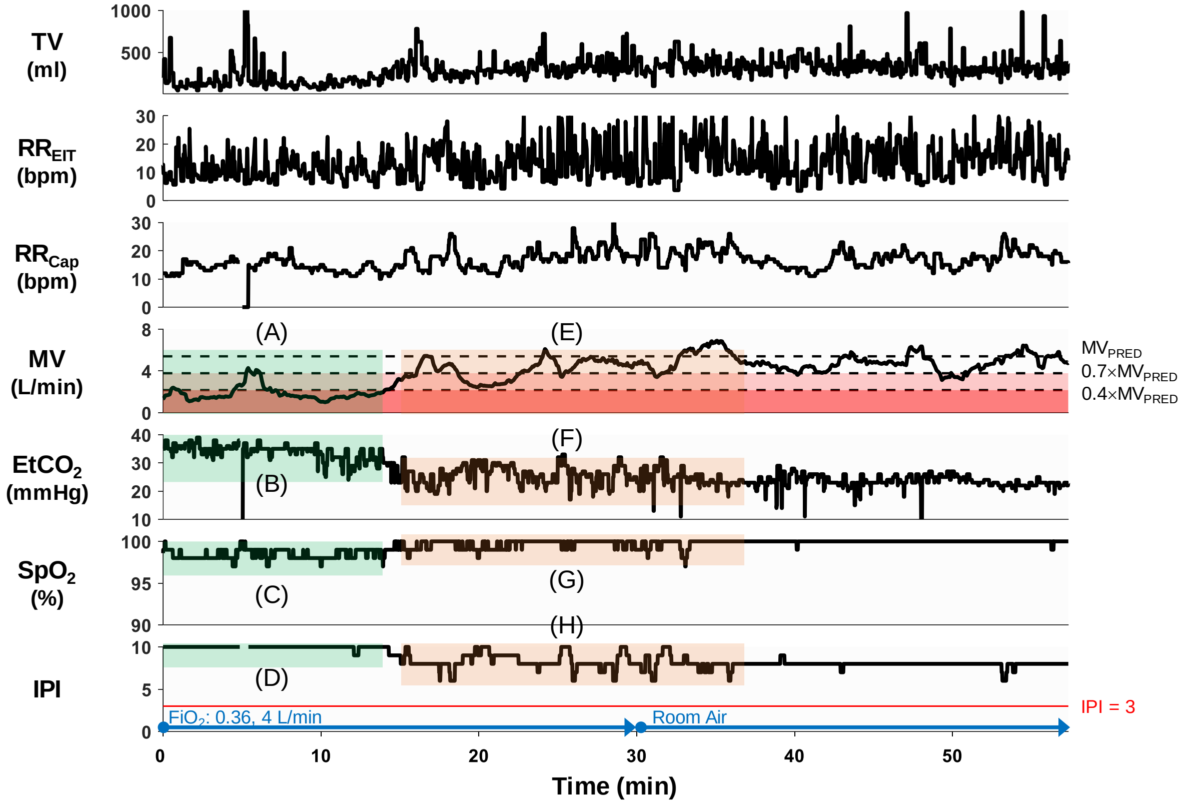

(A) MV was at times below $0.4 \times MV_{PRED}$.

(B) $EtCO_2$ was stable around 35 mmHg.

(C) SpO_2 was normal.

(D) IPI was either 9 or 10.

(E) MV increased and mostly stayed above $0.7 \times MV_{PRED}$.

(F) $EtCO_2$ decreased below 25 mmHg.

(G) SpO_2 was normal.

(H) IPI fluctuated between 5 and 10.

Figure 12. Measured data from the patient #12. The Matlab (MathWorks, U.S.) software was used to generate the plots.

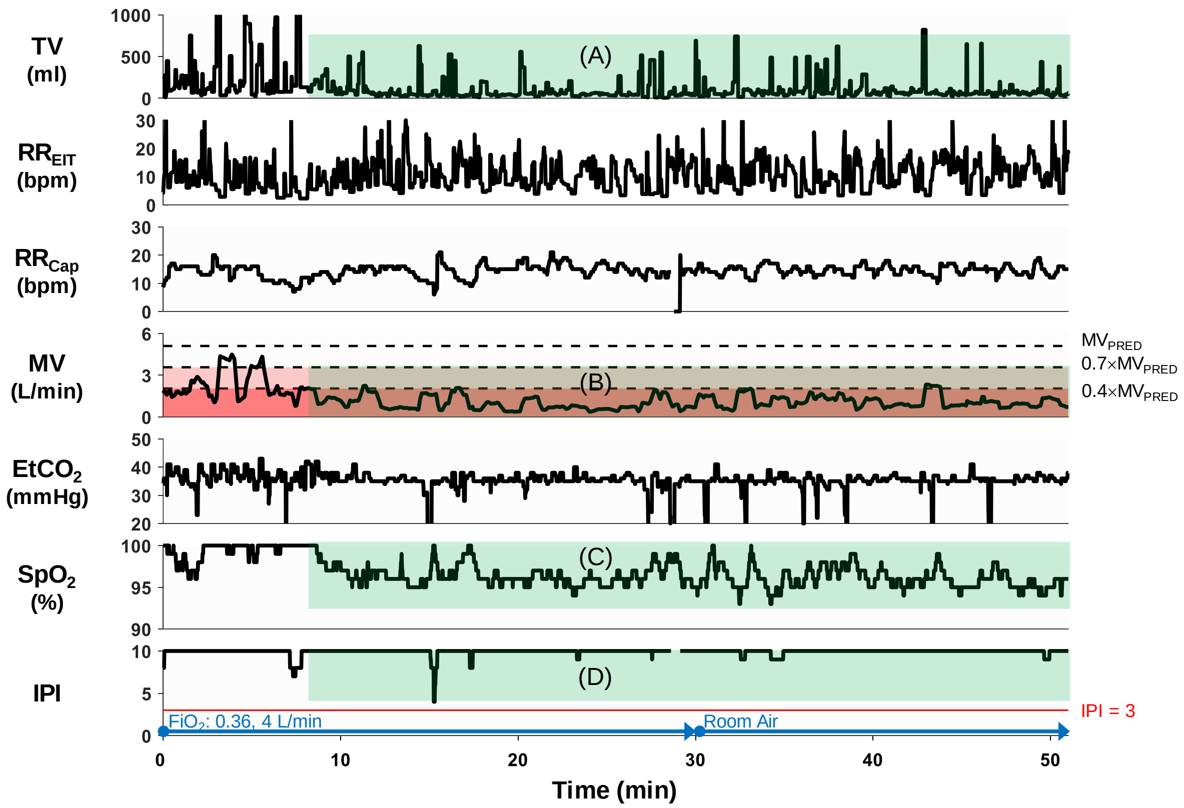

(A) TV was low with occasional deep breaths.

(B) MV was mostly below $0.4 \times MV_{PRED}$ (hypoventilation).

(C) SpO_2 fluctuated between 93% and 100% before and after switching to room air.

(D) IPI was normal.

Figure 13. Measured data from the patient #13. The Matlab (MathWorks, U.S.) software was used to generate the plots.
